# Patterns and rates of intron divergence between humans and chimpanzees

**DOI:** 10.1186/gb-2007-8-2-r21

**Published:** 2007-02-19

**Authors:** Elodie Gazave, Tomàs Marqués-Bonet, Olga Fernando, Brian Charlesworth, Arcadi Navarro

**Affiliations:** 1 Unitat de Biologia Evolutiva, Departament de Ciències Experimentals i de la Salut, Universitat Pompeu Fabra, Carrer Dr Aiguader 88, 08003 Barcelona, Catalonia, Spain; 2 Instituto de Tecnologia Química e Biológica (ITQB), Universidade Nova de Lisboa, Av. da República (EAN) 2781-901 Oeiras, Lisboa, Portugal; 3 Institute of Evolutionary Biology, University of Edinburgh, West Mains Road, Edinburgh, Scotland, EH7 3JT, UK; 4 Institucio Catalana de Recerca i Estudis Avancats (ICREA), Unitat de Biologia Evolutiva, Departament de Ciències Experimentals i de la Salut, Universitat Pompeu Fabra, Carrer Dr Aiguader 88, 08003 Barcelona, Catalonia, Spain

## Abstract

**Background:**

Introns, which constitute the largest fraction of eukaryotic genes and which had been considered to be neutral sequences, are increasingly acknowledged as having important functions. Several studies have investigated levels of evolutionary constraint along introns and across classes of introns of different length and location within genes. However, thus far these studies have yielded contradictory results.

**Results:**

We present the first analysis of human-chimpanzee intron divergence, in which differences in the number of substitutions per intronic site (K_i_) can be interpreted as the footprint of different intensities and directions of the pressures of natural selection. Our main findings are as follows: there was a strong positive correlation between intron length and divergence; there was a strong negative correlation between intron length and GC content; and divergence rates vary along introns and depending on their ordinal position within genes (for instance, first introns are more GC rich, longer and more divergent, and divergence is lower at the 3' and 5' ends of all types of introns).

**Conclusion:**

We show that the higher divergence of first introns is related to their larger size. Also, the lower divergence of short introns suggests that they may harbor a relatively greater proportion of regulatory elements than long introns. Moreover, our results are consistent with the presence of functionally relevant sequences near the 5' and 3' ends of introns. Finally, our findings suggest that other parts of introns may also be under selective constraints.

## Abstract 2

An analysis of human-chimpanzee intron divergence shows strong correlations between intron length and divergence and GC-content.

## Background

Introns are neither neutrally evolving sequences nor junk DNA, as they were once considered to be. Increasing amounts of evidence show that they harbor a variety of untranslated RNAs, including microRNAs, small nucleolar RNAs, and guide RNAs for RNA editing [1]. Introns are also important for mRNA processing and transport [2]. Moreover, microarray tiling experiments [3] have shown that a substantial part of the cell's transcriptional activity involves polyadenylated RNA that appears to be derived from intergenic regions, antisense sequences of known transcripts, and introns. Also, recent studies [4,5] show that almost all small nucleolar RNAs and a large proportion of microRNAs in animals are encoded in introns. Finally, novel intronic transcripts are continually being reported (for instance, see the report by Kampa and coworkers [6]), even though their functional properties are still largely unknown. This evidence implies that at least a fraction of intronic regions have functions and that they are likely to be evolving under the influence of natural selection, mostly purifying selection.

The effects of selective constraints on patterns of nucleotide divergence and polymorphism have been used by previous authors as a way to investigate the functional properties of introns. Several studies have been performed using *Drosophila *data. Marais and coworkers [7] showed that first introns are on average two times longer than other introns. They also found a negative correlation between protein divergence rates between *D. melanogaster *and *D. yakuba *and the lengths of introns in the corresponding genes. However, subsequent studies contradict those results. In a comparison of *D. melanogaster *and *D. simulans*, Haddrill and coworkers [8] found that first introns are not evolving more slowly or faster than other introns, whereas the class of long introns had higher GC content and lower divergence than short introns.

Evidence from mammalian introns is also contradictory. Various studies have demonstrated the presence of regulatory elements in mammalian introns, particularly first introns [9-11]. Also, in both mouse [12] and human [13], it has been shown that first introns enhance gene expression more than any others. If first introns were enriched with regulatory elements, they should thus have lower rates of evolution than other introns. Chamary and Hurst [14] showed that this is the case when comparing mouse and rat sequences. Consistent with this, Gaffney and Keightley [15] observed a negative correlation between mean intronic selective constraint and intron ordinal number, meaning that first introns are more conserved between rat and mouse than other introns. However, this contradicts a previous analysis [16] of divergence between human and mouse introns, which found that first introns evolve faster than other introns. Although these studies are difficult to compare because they use different pairs of species, the discrepancy remains puzzling. It may be attributed to difficult alignment of introns over the long evolutionary distances between human and mouse, or perhaps to different selective pressures acting in different lineages. Thus far, no clear resolution to this puzzle has been provided.

Among this confusing set of contradictory results, two undisputable facts about human introns emerge. First, human introns contain regulatory elements and splicing control elements that may affect patterns of genetic divergence. Second, first introns tend to be longer than introns in other positions of the gene [17,18]. Majewski and Ott [19] showed that, in humans, introns possess splicing control elements, at least within a distance of 150 nucleotides from intron-exon boundaries. They found that insertions of short interspersed repeats, microsatellite repeats, and the presence of single nucleotide polymorphisms were greatly reduced in such regions, especially in first introns. This suggests that these intron fragments are likely to be under purifying selection. Also, low complexity regions and simple repetitive elements are more abundant near intron-exon boundaries, suggesting a role in splicing regulation. Furthermore, human first introns are enriched in transcription regulatory elements, especially in the first 1,000 nucleotides from the intron-exon boundary at the 5' end [19].

We would expect that putatively regulatory intronic regions would be conserved between human and a closely related species such as chimpanzee. The availability of genome assemblies for both species offers the possibility to assess intron characteristics at the whole genome scale. Here, we investigate intron divergence patterns between these two species, as indicated by K_i _(the number of substitutions per nucleotide in introns), between truly orthologous pairs of human-chimpanzee introns. We describe the levels of molecular divergence between human and chimpanzee introns and show that these depend on characteristics such as intron length, order in the gene, and nucleotide composition. In addition, we propose that although the differences in size and rate of evolution among introns depend on many factors, they are mainly determined by their regulatory element content.

## Results

### Divergence, length, GC content, and CpG islands

Introns have an average human-chimpanzee divergence of 1.018% (measured as K_i_, the percentage of nucleotide changes per intron), a mean length of 3,219.59 nucleotides, and a mean GC content of 43.51%. The mean proportion of intron sequence represented by CpG islands is 2.71% (Table 1). A first analysis shows that intron divergence is positively correlated with GC content (*r *= 0.115, *P *< 10^-5^). Also, introns longer than the median of 1,029 nucleotides (defined as 'long' introns; see Materials and methods, below) are more divergent than short introns (K_i _= 0.974 versus K_i _= 1.061; Table 1). However, GC content correlates negatively with length (*r *= -0.107, *P *< 10^-5^). That is, long introns diverge more but they are poorer in GC content.

First introns are different from other introns; they are on average richer in GC content, longer, and diverge more than do other introns (Table 1). To determine whether first introns diverge more because of their length or because they are richer in GC content, we examined these relationships within each size class (short and long). The differences in divergence and GC content between first and nonfirst introns follow the same trends within the short and long intron classes (Table 2). Differences in GC content between first and nonfirst introns are almost equivalent for short and long introns. In contrast, divergence differences between first and nonfirst introns are clearly greater within the short category (Additional data file 2). This suggests that divergence differences between first and nonfirst introns are at least partly accounted for by factors related to their length rather than factors related to their nucleotide composition. To further tease out the possible confounding effect of GC content on the relationship between intron divergence and length in first introns, we conducted a nonparametric partial correlation analysis between length and divergence. The relationship between intron length and divergence remains after controlling for the effect of GC content (Spearman *r *= 0.138, *P *< 0.01).

Nevertheless, a relationship between GC content and divergence exists, suggesting that mutational biases may explain part of the divergence differences between intron classes. In mammals, nucleotide composition is correlated with the presence of CpG islands, whose relationship with divergence is unclear. To check whether the differential divergence between short and long and between first and nonfirst introns is associated with the presence of CpG islands, we measured the proportion of intron sequence constituted by these genomic features. Table 1 shows that first introns are tenfold richer in CpG islands than are other introns. This is also the case for short introns, which contain a four times greater proportion of CpG islands than long introns (long and first introns diverge more but they have, respectively, low and high CpG island coverage).

**Figure 1:**
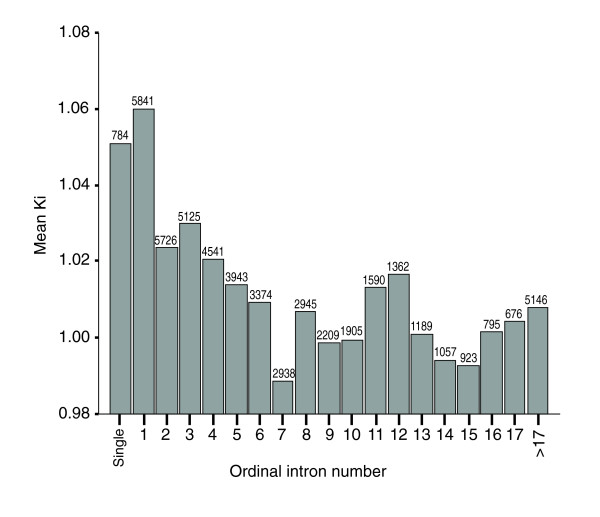
Mean K_i _as a function of the ordinal position of introns (relative to other introns of the same gene). Single introns constitute a special category. All introns whose number within the gene was above 20 were pooled together, to avoid classes of sample size that was too different. The number above each bar represents the sample size of each category. First and single introns are the more divergent ones.

**Figure 2:**
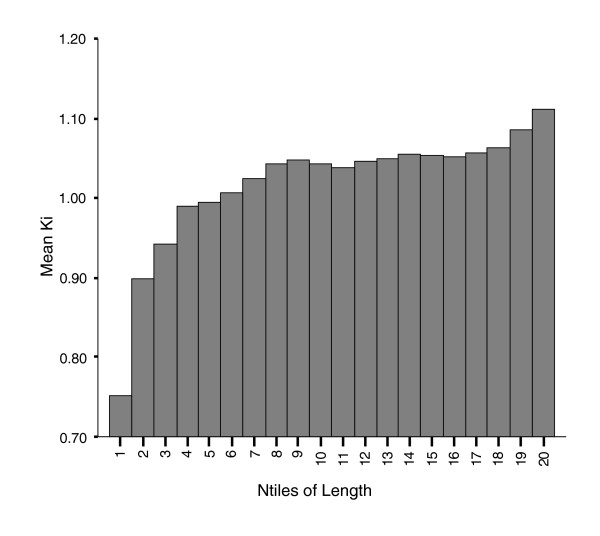
Average K_i _for 20 classes of percentiles of length. Although there is a global increase in divergence with size, the shortest class of size presents an especially low divergence compared with all of the following classes of intron size.

We also studied in detail the relationship between the ordinal position of introns in a gene (first intron, second intron, and so on) and divergence. The global correlation between intron order and K_i _is significant but very weak (*r *= -0.020, *P *< 10^-4^) and mostly due to first introns, because the correlation drops dramatically when they are removed (*r *= -0.010, *P *= 0.04). This indicates that divergence does not decay slowly and regularly with the ordinal position of introns in a gene, but that high average divergence is exclusive to first introns (Figure 1). Also, the relationship between intron length and K_i _is nonlinear. At first, there is a steep increase in divergence for the 35% shortest introns of the dataset (that is, the seven first classes of percentiles of length in Figure 2), followed by a higher homogeneity in divergence for larger introns (Figure 2). Because 35% is somewhat below the threshold that we used to define the class labeled as 'short' (median of the size distribution), we can say that the relationship between K_i _and length is especially strong for the shortest of short introns.

Finally, and as an additional way of ensuring that the higher divergence of first introns was not due to their higher average size, we separated them into 'long' and 'short' categories according to their median size. In this way, and only for this analysis, long first introns were those above 2,020 nucleotides and short first introns were those equal to or below this length. When comparing the 2,921 long and 2,920 short first introns classified according to this criterion, we observed that short first introns were significantly more conserved and significantly richer in GC content than were long first introns, following exactly the same trends as described above for nonfirst introns (K_i short _= 1.041, K_i long _= 1.079 [*P *= 0.0030]; GC _short _= 0.522, GC _long _= 0.425 [*P *< 10^-5^]). This therefore confirms an intrinsic length effect.

### Divergence, splicing control sites, and regulatory elements

To assess whether the greater divergence of long and first introns was related to their relative amount of regulatory elements, we performed some additional analyses. Introns possess splicing control elements in their 150 first 5' and 3' nucleotides from the intron-exon boundary [19]. Furthermore, human first introns are enriched in transcription regulatory elements, especially in their first 1,000 nucleotides at the 5' end [19]. Short introns may possess a greater proportion of such elements, thereby explaining their lower divergence.

To test this hypothesis, we divided all introns into three fragments: the first 150 nucleotides from the 5' end, the last 150 nucleotides from the 3' end, and the remaining central part. We also split first introns into three fragments: the first 1,000 nucleotides at the 5' end, the last 150 nucleotides at the 3' end, and the remaining part. Because all the comparisons on these fragments were performed on the unmasked dataset (see Material and methods, below), the raw values of K_i _and GC content cannot directly be compared with those of the analysis above. For example, the addition of repetitive elements has the effect of increasing the average K_i _value of the whole sample (K_i masked _= 1.043, K_i unmasked _= 1.142, *n *= 37,682; *P *< 0.001).

The regions that were previously shown to harbor splicing control sites (150 nucleotides at the 5' and 3' ends of all introns) diverge much less than the central part of the introns (Table 3). Furthermore, these highly conserved regions do not differ in K_i _between long and short introns (Table 3), supporting the hypothesis that they contain elements common to all introns, independent of their length. The central parts of all introns (what remains after removing the 150 nucleotides at the 5' ends and 150 nucleotides at the 3' ends) still exhibit greater divergence in long introns than in short ones. Low divergence of short introns is therefore not due only to a higher proportion of known splicing control elements in their boundaries. Also, the central parts of longer introns have lower GC contents (Table 3).

The 1,000 nucleotides at the 5' ends of first introns, potentially containing regulatory elements such as transcription factor binding sites [19,20], are also more conserved than the central part of first introns (Table 3). However, the difference in divergence for these 1,000 nucleotides between long and short first introns is marginally significant, in the opposite direction to what we observed for the 150 nucleotides in 5' ends of all introns (Table 3). That is, the first 1,000 nucleotides at the 5' end are more divergent in short than in long introns. This may mean that regulatory elements in short first introns are different from those in long first introns. However, we must be cautious with this interpretation, given the small sample size available for this test. This is because of the fact that the analysis above includes only the longest introns of the 'short' class (introns above 1,199 nucleotides), because we removed 1,000 + 150 nucleotides at both ends and we did not retain the central part when its size was less than 49 nucleotides (corresponding to the minimum intron size that we decided to include in the analysis). It is possible to have introns labeled as 'short' although they have a size above 1,199 nucleotides because we used the unmasked dataset for the analysis of intron fragments (see Material and methods, below, for more details). An alternative explanation would be that the conserved part of first introns does not span as much as 1,000 nucleotides. We can also see in Table 3 that, in the case of first introns, the difference in divergence between short and long introns after removing the 1,000 nucleotides at the 5' end is no longer significant. This suggests that, in contrast to other introns, divergence in first introns is independent of size, once the portion of their sequence composed by elements under very strong purifying selection is removed. Finally, when comparing the central part of all nonfirst introns with the central part of first introns alone, we see that first introns still diverge significantly more than other introns (Table 3). In other words, even after removing the outermost intron regions, where most constrained sequences are located, first introns are still characterized by higher divergence rates.

**Figure 3:**
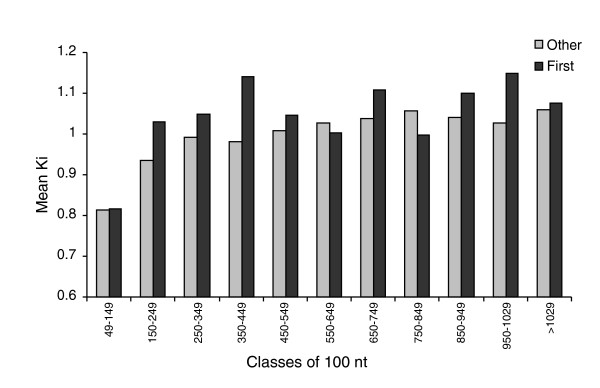
Evolution of K_i _within short introns (49 to 1029 nucleotides). The last bar of the histogram represents the cumulative data for all long introns. Data are presented for first and nonfirst introns separately, and are pooled in categories of increasing size class of 100 nucleotides for visual clarity. Nonfirst introns reach a plateau of mean K_i _around 300 nucleotides, whereas this pattern is not as clearly discernable in first introns. nt, nucleotides.

To further study the relationship between intron length and divergence, we divided introns into different categories of size, grouping them into intervals of 100 nucleotides. Figure 3 shows K_i _for introns of these different length classes. In the same figure, we can see that, after a steep increase, divergence seems to reach a plateau for introns of 300 nucleotides and more. This pattern looks less even for first introns than for other introns, perhaps because of lower sample size in each length class. This value of 300 nucleotides closely corresponds to the 150 nucleotides at the 5' ends plus the 150 nucleotides at the 3' ends that are probably under purifying selection. Introns of shorter size than 300 nucleotides mostly have highly conserved sequences. We can also see that, in the shortest class of introns (49-150 nucleotides), there is apparently almost no difference between first and nonfirst introns (Figure 3).

Finally, we wished to investigate whether introns of single-intron genes had special characteristics. We observe that single introns are significantly longer than the other introns. The difference in mean K_i _values between single and other introns is not significant, although the divergence of single introns is almost as high as that of first introns (K_i first _= 1.060, K_i single _= 1.051; Table 4 and Figure 1). Low sample sizes may account for the lack of significant results. If that were the case, then the high divergence of single introns could perhaps be explained by their size, but - as for first introns - an explanation for their length would still be needed.

Regarding variation in GC content among the different intron fragments, no consistent patterns were found. In some cases, higher GC is associated with higher K_i_, whereas in others the more divergent category is associated with the lowest GC content (Table 3).

### Housekeeping genes and divergence in intact introns

After removing the outmost parts of introns, which are putatively under stronger purifying selection than their central parts, we still observe lower substitution rates in short introns. This can be due either to an enrichment in conserved regulatory elements or to other factors that are correlated with length. Castillo-Davis and coworkers [21] showed that introns of housekeeping genes were shorter and richer in GC content. These patterns were also detected in our dataset. In addition, we found that introns of housekeeping genes are more conserved, although the difference is only marginally significant (Table 5). To determine whether the class of short introns diverges less because it is enriched in housekeeping genes, we removed housekeeping genes and repeated our long/short analysis. The difference between short and long introns is still significant (Table 5), meaning that the effect of housekeeping genes is not the only factor affecting the difference in evolutionary rates between introns of different lengths.

### Recombination

As expected, divergence and recombination are significantly correlated in the masked dataset (*r *= 0.118, *P *< 0.001), the correlation being observed in both short and long introns (*r*_short _= 0.083, *P *< 0.001; *r*_long _= 0.156, *P *< 0.001). We also confirm that recombination positively correlates with GC content (*r *= 0.175, *P *< 0.001). Finally, there is no overall correlation between intron length and recombination (*r *= 0.006, *P *= 0.255). When performed within each class of size (short and long), the correlations between recombination and length are significant, but their signs are different. That is, recombination rate does not have a linear relationship with length; it is negatively correlated with length for short introns (*r*_short _= - 0.045, *P *< 0.001), but positively correlated - albeit weakly - with length for long introns (*r*_long _= 0.014, *P *= 0.036).

Recombination rates are higher in first and in short introns (Table 6). That is, first introns recombine more, perhaps because - on average - they are longer. When focusing only on these, we observed the same pattern of variation between recombination and length as for the whole dataset, although correlations are not significant (*r*_short _= - 0.022, *P *= 0.436; *r*_long _= 0.003, *P *= 0.854).

### Known evolutionary factors affecting sequence divergence

Some of the analyses presented above might have been biased by factors that are known to affect rates of divergence and/or intron length. For example, if genes in the X chromosome had shorter and less divergent introns, then this could artefactually give rise to some of the patterns we detected. To ensure that this is not the case, we repeated our main tests after controlling for these factors (see Material and methods, below, and Additional data file 1). This analysis revealed a few biases, some of which are conservative (they go in the opposite direction to our overall results). For example, introns of chromosome 19, which are highly divergent, tend to be shorter than introns elsewhere in the genome. Also, introns located in telomeres and centromeres are shorter than introns outside these regions but, in contrast, divergence rates go in opposite directions, being higher in telomeres and lower in centromeres (Additional data file 1). At any rate, our results remain the same after removing genes located in these regions, meaning that introns of different classes are equally affected by these factors. This indicates that the differences in divergence between short and long introns that we reported above are not due to a higher proportion of certain intron classes in given chromosomes or genomic regions.

## Discussion

The overall picture that emerges from our findings is that, as revealed by human and chimpanzee divergence, different introns and different parts of introns may have been subjected to different evolutionary forces, among which is natural selection. Our first series of results are related to intron length and nucleotide composition, showing a negative correlation between intron size and GC content. A steep decrease in GC content with intron length had previously been reported in the human genome [18]; in contrast, no such relationship has been reported for exon length. Moreover, Majewski and Ott [19] showed that first introns have the striking feature of being the most GC-rich elements of a gene, with an average GC content up to 65% near the 5' splicing site. According to those authors, this pattern is due to an over-abundance of regulatory motifs such as CpG and GGG trinucleotides. In the same study, an excess of CCC triplets was found near both splice sites, whereas other dinucleotides or trinucleotides did not exhibit such effects. Finally, G-rich elements have been shown to act as splicing enhancers [22]. Majewski and Ott [19] also emphasized that the internal parts of introns do not exhibit an excess of CpG. The global GC enrichment that we found in first introns compared with other introns may thus reflect their higher density of GC-rich regulatory elements. We observed that the categories with a higher GC content are enriched in CpG islands, which is consistent with results from previous authors (see, for example, Takai and Jones [23]). CpG islands are frequently associated with the 5' ends of genes and are thought to play an important role in the regulation of gene expression [24]; this may explain their abundance in first introns.

Another series of results involves patterns of divergence. GC content is positively correlated with intron divergence. However, as mentioned above, intronic regulatory sequences are expected to be enriched in GC. Therefore, the higher divergence of GC-rich introns may seem paradoxical, because we would expect GC-rich regulatory motifs to be selectively constrained. However, the positive correlation between intron size and divergence that we detected suggests that the density of conserved sequences is lower in long introns. This may explain why long introns are, simultaneously, GC poorer and more divergent. A class of constrained sequences that could account for this effect are splicing control sites, located close to exon-intron boundaries. However, after removing the outmost 150 nucleotides at both ends of all introns, divergence is still lower in short introns, so their relative higher density of splicing control sites cannot explain the positive correlation between intron size and divergence.

Thus, other factors need to be invoked to explain the lower divergence of short introns. First of all, it is possible that other classes of regulatory elements, in particular not GC-based motifs, that we did not take into account are distributed all over the introns, and are not only located in the 150 nucleotides close to intron-exon boundaries. This would be consistent with previous experimental work describing some such elements [25,26]. If this were the case, then short introns would diverge less because of their relatively higher proportion of regulatory elements.

As mentioned above, CpG islands are associated with gene expression regulation. They are also constitutively hypomethylated, and lack the mutagenic effect seen in their methylated CpG counterparts [27]. We found that short introns contain a higher proportion of CpG islands, which could account for their lower divergence compared with long introns. However, first introns are more divergent than other introns, and also have a much higher density of CpG islands than nonfirst introns. In summary, a higher density of CpG islands is found in both slowly diverging short introns and rapidly diverging first introns. This suggests that CpG islands do not have a direct overall effect upon rates of divergence in introns.

A potential factor directly linking intron length and divergence is recombination. In agreement with previous studies [28,29], we found that length is negatively correlated with GC content in human introns; divergence and GC content are both positively correlated with recombination rate. Still, the correlations we detected are too weak to have any biologic relevance; also, the fact that in the human genome most recombination takes place in hotspots separated by an average distance of 200 kilobases [30] may be artefactually inflating recombination in long introns compared with shorter ones. Recombination thus does not seem able to explain our results.

Another hypothesis to explain the relationship between size and divergence in our data is that the class of short introns is enriched in introns from housekeeping genes, because introns are substantially shorter [31] and GC richer [21] in such highly expressed genes. The shorter size of introns in housekeeping genes has been suggested to reflect the influence of strong selective pressures to reduce their transcriptional cost [21]. This hypothesis is referred to by some authors as the 'selection for economy' hypothesis, and implicitly assumes a neutralist interpretation of the accumulation of DNA in eukaryotic genomes. However, even if the introns of housekeeping genes are indeed less divergent, GC richer, and shorter, our results remain the same after removing them, suggesting that the 'selection for economy' model cannot explain intron evolution on its own. In a recent report, Vinogradov [32] tested alternative hypotheses to explain variations in intron size within the genome. In particular, he investigated the adaptationist 'genome design' hypothesis, which proposes that the intragenic and intergenic noncoding DNA, in which tissue specific genes are embedded, is involved in regulation. In other words, the variation in length of genomic elements such as introns is determined by their function. Elements such as transcription factor binding sites and noncoding RNAs present in introns may be in a higher proportion in development-specific and condition-specific genes, which need fine and very complex regulation, and would thus have longer introns than housekeeping genes. Vinogradov [32] found a strong relationship between the length of conserved intronic sequences between human and mouse and the number of functional domains in the corresponding proteins, and therefore favored the 'genome design' model over the 'selection for economy' one. The results on *Drosophila *reported by Haddrill and coworkers [8] also support this model, even though they differ from our findings in other aspects, as discussed below.

Many studies have shown that selectively constrained noncoding DNA and intron-associated control elements are more frequently found in first introns than other introns [9-11,20], especially close to the 5' end of first introns [19] or close to the start codon [33]. Again, it may seem contradictory that first introns harbor more regulatory and control elements and are simultaneously more divergent than other introns. However, as underlined by Chamary and Hurst [14], the fact that first introns are longer and harbor a higher number of regulatory elements does not imply that their overall density of constrained sites is higher. For example, if an interaction between transcription factor binding sites with chromatin structure is necessary for correct transcriptional regulation, as suggested by Vinogradov [32], then a minimum spacing between these binding sites might be required. This would explain why first introns are on average longer than other introns. Unfortunately, this hypothesis is difficult to test because regulatory motifs are short sequences of low informational content [34,35], so that most of them are still unknown or difficult to differentiate from spurious sequences.

Thus far we have tried to describe the patterns of intron divergence between humans and chimpanzees, and to propose hypotheses regarding the forces that act on intron evolution, comparing our results to findings from other species. In many cases, these results are contradictory to ours. An example of such contradiction is the positive correlation between GC content and divergence that we report here, which is in conrast to the results reported by Haddrill and coworkers [8] on *Drosophila*. Apart from the fact that the difference in distribution of intron size between *Drosophila *and human/chimpanzee makes it difficult to compare the two sets of findings (Additional data file 3), the discrepancy must be somehow related to the fact that forces acting on nucleotide composition are very different in different lineages. Indeed, Aerts *et al*. [36] detected opposite changes of relative AT richness in humans and flies around transcription start sites, proposing that fly genes differ from humans in their AT content because of differences in their concentration of AT-rich transcription factor binding sites around transcription start sites. Another example also comes from the analysis conducted by Haddrill and coworkers [8]. These authors provided evidence that variation in GC content may reflect local variation in mutational rates or biases, or the effects of biased gene conversion favoring GC over AT, which mimics selection in favor of GC dinucleotides. However, in a study of mouse-rat genome divergence, Chamary and Hurst [14] showed that transcription-coupled mutational processes and biased gene conversion cannot explain sequence evolution. Rather, they presented strong evidence for selectively driven codon usage in mammals.

A further example of contradictory data coming from different species is reported by Presgraves [37]. In that study of the pattern of small insertions and deletions in different *Drosophila *species, Presgraves suggested that intron length evolution is affected by chromosome-specific and lineage-specific forces. Using *Drosophila yakuba *as an outgroup, he showed that in *D. melanogaster *X-linked introns have slightly increased in size, whereas autosomal ones have slightly decreased in size. In contrast, in *D. simulans *both autosomes and the X chromosome have decreased in size since their divergence from *D. yakuba*. Presgraves' conclusion was that this observation could not easily be explained by a single general model of intron length. These examples highlight the difficulties in comparing modes of intron evolution between distant groups of species. If such different trends can be observed in sister species, then it is only to be expected that results between more distant species are even more dissimilar. In the data analyzed here, we found X chromosome introns to be shorter and more divergent than autosomal introns. Comparing pairs of paralogous introns in the human genome, Cardazzo and coworkers [38] also found that introns of autosomal genes are significantly longer than X-linked introns. Therefore, although our results are not identical to those with other species, they are at least consistent with previous studies on the lengths of human introns.

The importance of functional elements in noncoding sequences of the genome is becoming increasingly acknowledged. Conserved noncoding sequences have been shown to be selectively constrained [39]. Among the 327,000 conserved nongenic sequences that were recently found in the human genome, 35% were located in introns (for review, see Dermitzakis and coworkers [40]). Bejerano and colleagues [41] showed that around 100 of the 481 ultraconserved elements in the human genome (that is, sequences having 100% similarity between human and mouse and stretching over = 200 nucleotides) map within introns. Although the functions of these noncoding conserved sequences is mostly unknown, at least some of them play a regulatory role [42]. Until now, only very few studies have evaluated the action of selection on noncoding regions through the study of their divergence levels among species [34,43,44]. This confirms that selection is acting on upstream regions of genes [34,43] and 5'-untranslated regions [45]. However, to our knowledge, no study has yet been performed on introns. Such an analysis is currently underway.

## Conclusion

We showed that, even after correcting for some potentially confounding factors, long introns have higher divergence between humans and chimpanzees than short introns, whereas GC content and length are negatively correlated. Another pattern is that divergence rates are higher in first introns than in nonfirst introns. The higher divergence of first introns is partly related to their longer length. This may reflect a high proportion of functional elements distributed along their sequence, separated by unconstrained regions. Finally, we also show that the 5' and 3' ends of introns, which are known to contain regulatory elements and splicing control sites, have lower divergence than the remaining parts of introns. The best explanation for all these patterns is that purifying selection has a strong effect on shaping intron sequence evolution. It is also possible that divergence patterns and rates between human and chimpanzee introns have been affected by positive selection. To follow up this work, the next step would be to determine whether this is true and, if so, to identify which are the introns that may have undergone positive selection.

## Materials and methods

### Sequence gathering and alignment

We generated a first dataset, composed of sequences obtained from the RefSeq database [45]. The sequences correspond to human genome build 35 and chimpanzee genome build 1. Human intron positions were obtained from the UCSC Human RefSeq table [46]. Sequences were gathered from the masked human genome and their corresponding chimpanzee sequences were obtained from the positions of UCSC XenorefSeq table [46].

To avoid biases introduced by misalignment, every individual intron was aligned with the local alignment tool BLAST 2 Sequences [47], which uses the BLAST algorithm with default parameter values. In contrast to global alignment tools, such as CLUSTALW, local alignments do not force alignment between two sequences; if no good alignment is possible, the algorithm does not return any output. This allowed us to exclude a large number of false orthologous introns from our analysis. All local alignments for a given intron were joined by removing any overlapping parts (that is, locally aligning several times). To further avoid false intron orthology, we removed from the analysis any aligned intron pair for which less than 80% of the shortest sequence aligned to the other species. Also, we filtered out any genes with a different number of introns in the two species. Finally, because alternative splicing and multiple transcripts allow for sets of overlapping introns, we only kept the longest intron from each set. This produced a final intron dataset of 52,646 introns, corresponding to 7,791 genes.

To perform some analyses and comparisons (such as the exact determination of intron-exon boundaries), a second, nonmasked dataset was obtained by gathering the unmasked sequences of any intron that had passed the filtering process above.

### Divergence, GC content, and housekeeping genes

For every intron, human-chimpanzee divergence was measured applying the Jukes-Cantor correction to the number of substitutions per intronic site, K_i_, using the distmat application from the EMBOSS package [48]. Although we tried to exclude poorly aligned sequences, the dataset still contained some exceedingly high K_i _values, most likely due to false orthology assignments. The dataset was therefore filtered by removing all K_i _values three standard deviations above the mean. The filter was applied to the masked dataset and any intron with K_i _more than 3.025% was removed from both the masked and the unmasked datasets. This implies that some introns in the unmasked dataset may have higher divergence than this 3.025% limit if their masked version had a divergence below this threshold.

After this process, a total of 51,674 nonredundant introns was left, for which we know their order in the gene, length, GC content, and level of divergence between humans and chimpanzees. Their size in humans varies from 49 to 955,099 nucleotides and from 49 to 592,440 nucleotides after removing the masked repetitive elements. In chimpanzees, intron size varies from 49 to 974,461 nucleotides, and from 49 to 566,414 nucleotides after removing masked repetitive elements.

Ancestral intronic GC content was also estimated for every gene as the average of the current human and chimpanzee GC content. The positions of CpG islands were downloaded from the CpG island UCSC annotation database [46]. The overlap between each individual intron and CpG islands is expressed as a percentage of the total size of the masked introns. Recombination data were obtained from UCSC SNP Recombination Rates table [46]. All recombination values are given in centi-Morgans per megabase (cM/Mb). The list of housekeeping genes used in this paper is the one given by Hsiao and coworkers [49].

### Intron fragments

To study the divergence and GC content measurements in fragments of introns that are of particular interest (such as the first 150 nucleotides at the 5' and 3' boundaries of introns, where splicing control sites have been reported), a set of PERL scripts was written to cut up introns into fragments and measure their divergence and GC content. Because we were interested in these regions and because most known regulatory elements are composed of repetitive sequences, we performed this part of the study on the unmasked dataset. Also, to make sure that we were not losing regulatory elements, we only kept for analysis introns for which the alignment started between nucleotides 1 to 15 from the exon-intron boundary. Introns for which alignment started beyond that boundary were removed from this part of the analysis. After computation of K_i _for each segment independently (150 nucleotides at the 5' end, central part, and 150 nucleotides at the 3' end), all intron fragments with K_i _above 3.025 were again filtered out to make the new file similar to the one of the global sample. For the same reason, all the fragments of the central part (that is, after removing the 150 nucleotides at the 5' end and the 150 nucleotides at the 3' end, or 1,000 nucleotides at the 5' end and 150 nucleotides at the 3' end) that were less than 49 nucleotides long (corresponding to the minimum length of alignment on the global dataset) were removed from the analysis.

### Short and long introns

Introns were classified into two categories according to size. We followed the criteria used by Haddrill and coworkers [8], who defined introns as 'short' or 'long' based on the median of the length distribution. All introns shorter or equal to the median value (1,029 nucleotides) are labeled as 'short', and longer introns are labeled as 'long'. These categories were established in the masked dataset and, for consistent comparisons, the same short/long classification was kept for the unmasked dataset, even if lengths vary slightly between the two sets.

### Test of common evolutionary factors affecting molecular evolution

To study factors that are known to affect divergence rates, introns were further classified into five categories according to their genomic location: within the sex chromosomes, human chromosome 19, telomeres (10 Mb from both ends of the chromosome in either species), centromeres (5 Mb from the centromeres in either species), and/or segmental duplications (as defined in the Segmental Duplication Database [50]).

### Statistical tests

Divergence, length, and GC content were compared among introns belonging to different ordinal categories (for example, first versus nonfirst intron) or different classes of length (that is, short versus long introns) by means of pairwise permutation tests (based on 1,000 permutations, or 5,000 permutations when the *P *values obtained after 1,000 permutations were above 0.01). *P *values are the proportion of times that the difference in the averages of two categories in a permuted dataset was equal or larger than the observed difference. Correlation tests (Pearson's product-moment correlations) were performed with R, version 1.9.0 [51]. Nonparametric partial correlations were performed, as described by Haddrill and coworkers [8].

## Additional data files

The following additional data are available with the online version of this paper. Additional data file 1 is a table listing the mean K_i _and mean lengths for the main known factors affecting divergence rates. Additional data file 2 is a figure representing the mean GC content and mean K_i _for short and long introns. Additional data file 3 shows the comparative distribution of intron length between human and *Drosophila*. Additional data file 4 represents the Human RefSeq of the introns included in the analysis, with the main factors and variables we study here: K_i_, GC, first, and length (lengths are given for the sequences after masking).

## Supplementary Material

Additional data file 1 A table listing the mean K_i _and mean lengths for the main known factors affecting divergence rates. Click here for file

Additional data file 2 A figure representing the mean GC content and mean K_i _for short and long introns. Click here for file

Additional data file 3 Comparative distribution of intron length between human and *Drosophila*. Click here for file

Additional data file 4 Human RefSeq of the introns included in the analysis, with the main factors and variables we study here: Ki, GC, first, and length (lengths are given for the sequences after masking). Click here for file

## Figures and Tables

**Table 1:** K_i_, GC, CpG and length measures for all introns

	*n*	Variable	Mean	*P*
All introns	51,673	K_i_	1.018	-
All introns	51,673	GC	0.435	-
All introns	51,673	Length	3219.6	-
Short	25,849	Ki	0.974	
Long	25,824	Ki	1.0611	< 0.001
Short	25,849	GC	0.470	
Long	25,824	GC	0.401	< 0.001
First	5,841	K_i_	1.060	
Others	45,832	K_i_	1.012	< 0.001
First	5,841	GC	0.474	
Others	45,832	GC	0.430	< 0.001
First	5,841	Length	6971.7	
Others	45,832	Length	2741.4	< 0.001
First	5,841	CpG	12.48	
Others	45,832	CpG	1.47	< 0.001
Short	25,849	CpG	4.45	
Long	25,824	CpG	0.97	< 0.001

Shown are results of permutation tests between short and long introns and between first and other introns.

**Table 2:** Short versus long and first versus non-first introns

	Short introns					Long introns				
	
	N	Ki	P	GC	P	N	Ki	P	GC	P
First	1,880	1.028		0.550		3,961	1.075		0.438	
Others	23,969	0.970	< 0.001	0.463	< 0.001	21,863	1.059	0.016	0.339	< 0.001

Shown is a comparison of mean K_i _and GC content for first and other introns, within short introns, and within long introns.

**Table 3:** Intron fragments

	*n*	Variable	Mean	*P*
150 Nucleotides at 5' end versus central part of all introns				
5' end	36,384	K_i_	0.938	
Central	36,289	K_i_	1.144	< 0.001
5' end	36,384	GC	0.441	
Central	36,289	GC	0.432	< 0.001

150 Nucleotides at 3' end versus remainder of all introns				
3' end	36,456	K_i_	0.924	
Central	36,289	K_i_	1.144	< 0.001
3' end	36,456	GC	0.410	
Central	36,289	GC	0.432	< 0.001

1000 Nucleotides at 5' end versus central part of first introns				
5' end	3,295	K_i_	1.096	
Central	3,306	K_i_	1.195	< 0.001
5' end	3,295	GC	0.499	
Central	3,306	GC	0.435	< 0.001

150 Nucleotides at 5' end of all introns				
Short	14,892	K_i_	0.942	
Long	21,492	K_i_	0.935	0.371 (NS)
Short	14,892	GC	0.459	
Long	21,492	GC	0.429	< 0.001

150 Nucleotides at 3' end of all introns				
Short	14,929	K_i_	0.924	
Long	21,527	K_i_	0.924	0.991 (NS)
Short	14,929	GC	0.441	
Long	21,527	GC	0.389	< 0.001

5' 1000 Nucleotides of first introns				
Short	150	K_i_	1.193	
Long	3,145	K_i_	1.092	0.011
Short	150	GC	0.549	
Long	3,145	GC	0.499	0.234 (NS)

Central part after removing the 150 nucleotides at 5' and 3' end of all introns				
Short	14,014	K_i_	1.078	
Long	22,275	K_i_	1.185	< 0.001
Short	14,014	GC	0.451	
Long	22,275	GC	0.420	< 0.001

Central part after removing the 1000 nucleotides at 5' end of first introns				
Short	140	K_i_	1.172	
Long	3,166	K_i_	1.196	0.570 (NS)
Short	140	GC	0.457	
Long	3,166	GC	0.434	< 0.001

Central part of first introns versus central part of other introns				
First	3,306	K_i_	1.195	
Others	32,012	K_i_	1.140	< 0.001
First	3,306	GC	0.435	
Others	32,012	GC	0.429	< 0.001

Shown are the average K_i _and GC for different fragments of introns. NS, not significant.

**Table 4:** Single introns

	*n*	Variable	Mean	*P*
Single	784	Length	6253.5	
Others	50,889	Length	3172.8	< 0.001
Single	784	GC	0.486	
Others	50,889	GC	0.443	< 0.001
Single	784	K_i_	1.051	
Others	50,889	K_i_	1.017	0.086

Shown are the average length, GC content, and K_i _for single introns versus other introns.

**Table 5:** Housekeeping genes

	*n*	Variable	Mean	*P*
All introns				
Housekeeping	1129	Length	1513.4	
Others	50,544	Length	3257.7	< 0.001
Housekeeping	1,129	GC	0.450	
Others	50,544	GC	0.435	< 0.001
Housekeeping	1,129	K_i_	0.984	
Others	50,544	K_i_	1.018	0.037

Without housekeeping genes				
Short	25,116	K_i_	0.975	
Large	25,428	K_i_	1.061	< 0.001
First	5,689	Length	7083.7	
Others	44,855	Length	2772.5	< 0.001

Shown are the mean length, GC content and K_i _for housekeeping genes versus other genes. Also shown are mean K_i _and length for short versus large introns, and first versus other introns in all introns without housekeeping genes.

**Table 6:** Recombination

	*n*	Variable	mean	*P*
First	4,943	R	1.204	
Others	27,925	R	1.035	< 0.001
Short	10,895	R	1.116	
Large	21,973	R	1.033	< 0.001

Comparison of mean recombination rate, measured in cM/Mb, for first and other introns.
