# Supplementary material for: Patterns and rates of intron divergence between humans and chimpanzees
Source: Genome Biol. 2007 Feb 19;8(2):R21. doi: 10.1186/gb-2007-8-2-r21 (PMC1852421; doi:10.1186/gb-2007-8-2-r21)
Supplement: Additional data file 1 — A table listing the mean Ki and mean lengths for the main known factors affecting divergence rates. [file gb-2007-8-2-r21-S1.doc]

**Additional Table A1. Known factors affecting divergence rates.** Data represent, in the following order: Mean K_i_ for introns in different chromosomes and regions; Mean length for introns in different chromosomes and regions; Difference between short and long introns for all introns after removing the indicated factors, i.e. without X chromosome, i.e. without chromosome 19, i.e. without telomeres, i.e. without centromeres.

| ***Location of introns*** | **N** | **Variable** | **Mean** | **P** |
| --- | --- | --- | --- | --- |
| X chromosome | 478 | K_i_ | 0.771 |  |
| **Others** | 51194 | K_i_ | 1.020 | <0.001 |
|  |  |  |  |  |
| Y chromosome | 18 | K_i_ | 1.651 |  |
| **Others** | 51655 | K_i_ | 1.017 | <0.001 |
|  |  |  |  |  |
| **Chromosome 19** | 1958 | K_i_ | 1.160 |  |
| **Others** | 49715 | K_i_ | 1.012 | <0.001 |
|  |  |  |  |  |
| **Telomeres (10Mb)** | 7649 | K_i_ | 1.273 |  |
| **Non-telomeres** | 44024 | K_i_ | 0.973 | <0.001 |
|  |  |  |  |  |
| **Centromeres (+ 5Mb)** | 3323 | K_i_ | 0.933 |  |
| **Non-centromeres** | 48350 | K_i_ | 1.023 | <0.001 |

| ***Location of introns*** |  |  |  |  |
| --- | --- | --- | --- | --- |
| **X chromosome** | 478 | Length | 2348.9 |  |
| **Others** | 51194 | Length | 3227.7 | n.s. 0.067 |
|  |  |  |  |  |
| Y chromosome | 18 | Length | 1572.7 |  |
| **Others** | 51655 | Length | 3220.2 | n.s. 0.347 |
|  |  |  |  |  |
| **Chromosome 19** | 1958 | Length | 1027.8 |  |
| **Others** | 49715 | Length | 3305.9 | <0.001 |
|  |  |  |  |  |
| **Telomeres (10Mb)** | 7649 | Length | 2501.8 |  |
| **Non-telomeres** | 44024 | Length | 3344.3 | 0.001 |
|  |  |  |  |  |
| **Centromeres (+ 5Mb)** | 3323 | Length | 2644.7 |  |
| **Non-centromeres** | 48350 | Length | 3259.1 | <0.001 |

| ***Without chromosome X*** | | | | |
| --- | --- | --- | --- | --- |
| Short | 25586 | K_i_ | 0.977 |  |
| **Long** | 25609 | K_i_ | 1.063 | <0.001 |
| ***Without chromosome 19*** | | | | |
| **Short** | 24397 | K_i_ | 0.965 |  |
| **Long** | 25318 | K_i_ | 1.058 | <0.001 |
| ***Without telomeres*** | | | | |
| **Short** | 21536 | K_i_ | 0.925 |  |
| **Long** | 22488 | K_i_ | 1.020 | <0.001 |
| ***Without centromeres*** | | | | |
| **Short** | 23975 | K_i_ | 0.982 |  |
| **Long** | 24375 | K_i_ | 1.065 | <0.001 |
