# Supplementary material for: Patterns and rates of intron divergence between humans and chimpanzees
Source: Genome Biol. 2007 Feb 19;8(2):R21. doi: 10.1186/gb-2007-8-2-r21 (PMC1852421; doi:10.1186/gb-2007-8-2-r21)
Supplement: Additional data file 2 — A figure representing the mean GC content and mean Ki for short and long introns. [file gb-2007-8-2-r21-S2.pdf]

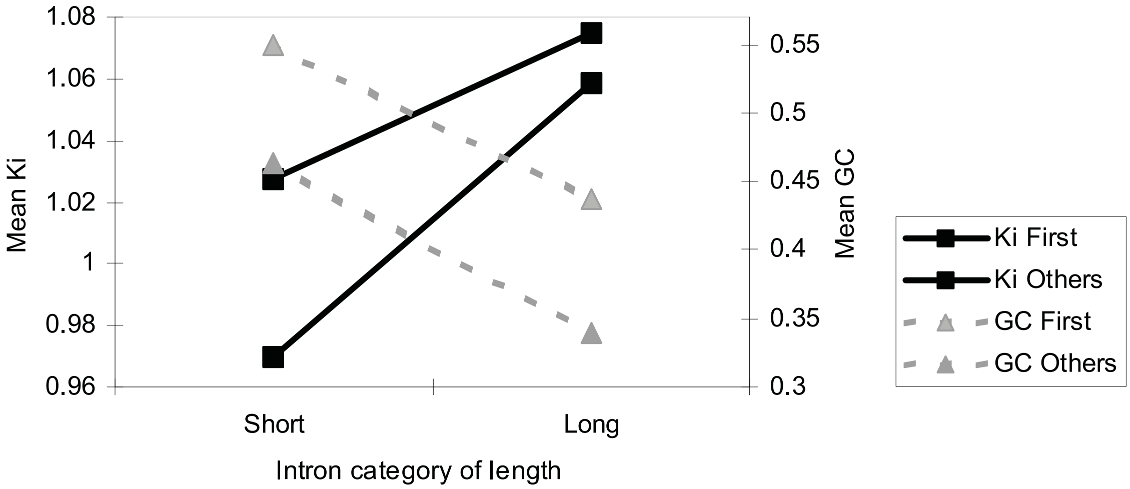

Additional data file 2. Mean GC content and mean K_i for short and long introns. GC content is different between first and non-first introns, but the difference remains the same within the short and the long intron classes. This is not the case for K_i where most of the difference in divergence between first and non-first introns is due to short introns.
