# Supplementary material for: Patterns and rates of intron divergence between humans and chimpanzees
Source: Genome Biol. 2007 Feb 19;8(2):R21. doi: 10.1186/gb-2007-8-2-r21 (PMC1852421; doi:10.1186/gb-2007-8-2-r21)
Supplement: Additional data file 3 — Comparative distribution of intron length between human and Drosophila. [file gb-2007-8-2-r21-S3.pdf]

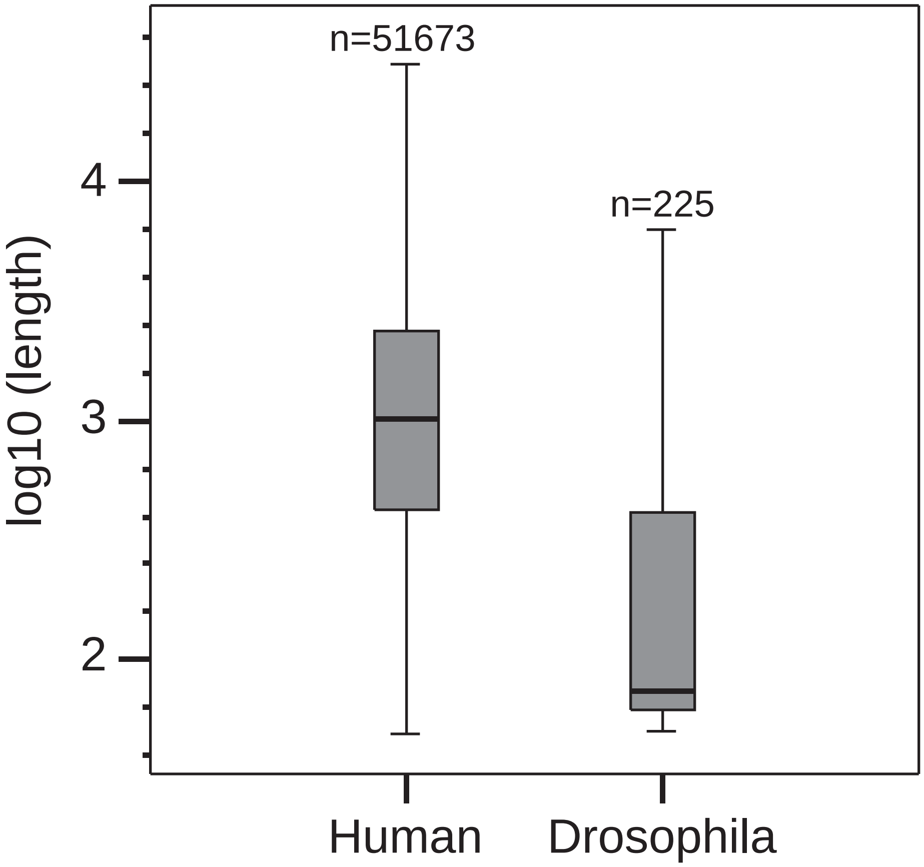

Additional data file 3. Comparative distribution of intron length between human and drosophila. The human data correspond to the present analysis, drosophila data are those used in Haddrill et al. [8] and were kindly provided by the authors. The bar in the middle of the box represents the median, the box defines the 25% and 75% quartiles, and the whiskers determine the upper and lower extremes of the length distribution. Values of length are log10 transformed for clarity.
